# Mitotic Activity, Cell Survival, and Neuronal Differentiation in the Hilus of the Dentate Gyrus Under Physiological and Hypothyroid Conditions in Adult Wistar Rats

**DOI:** 10.3390/cells14141112

**Published:** 2025-07-19

**Authors:** Karla Sánchez-Huerta, Ana Karen García-Juárez, Lía Diana Colmenero-Rodríguez, Yuliana García-Martínez, Jorge Pacheco-Rosado

**Affiliations:** 1 Laboratorio de Neurociencias, Subdirección de Medicina Experimental, Instituto Nacional de Pediatría, Insurgentes Sur 3700, Letra C, Alcaldía Coyoacán, Mexico City 04530, Mexico; ksanchezh@pediatria.gob.mx (K.S.-H.); ygarciamart@hotmail.com (Y.G.-M.); 2 Laboratorio de Neurofisiología, Departamento de Fisiología “Mauricio Russek”, Instituto Politécnico Nacional, ENCB, Prol. de Carpio y Plan de Ayala, s/n, Alcaldía Miguel Hidalgo, Mexico City 11340, Mexico; kate.garcia12@gmail.com (A.K.G.-J.); azulita_2tone@hotmail.com (L.D.C.-R.)

**Keywords:** granular neurons, hippocampus, hypothyroidism, methimazole, neuronal differentiation

## Abstract

The adult rodent hippocampus is capable of maintaining its capacity to generate new neurons in the subgranular zone (SGZ) of the dentate gyrus (DG). Interestingly, proliferative cells have also been described in the hilus. The involvement of the hilar neurogenesis process in hippocampal physiology is unknown. Thyroid hormones (THs) are necessary for the survival of postmitotic progenitor cells, neuroblasts, and immature granule neurons in the SGZ. In contrast, evidence concerning the role of THs in the hilar neurogenesis process is limited. The present study characterized the mitotic activity, cell survival, and neuronal differentiation of hilar neurogenesis under physiological and hypothyroid conditions and compared them with those of the granular layer (GL) and the SGZ of the DG in adult Wistar rats. We found that, under physiological conditions, the hilus harbors fewer proliferative cells than the neurogenic zone (GL/SGZ) does, with a rate of cell survival of 18.9% and a rate of differentiation into granular neurons of 19%. Interestingly, hypothyroidism provokes decreased cell proliferation and an increased rate of cell survival without affecting neuronal differentiation. These effects induced by hypothyroidism in the hilus were different or inclusive, contrary to those observed in the neurogenic zone.

## 1. Introduction

Neurogenesis in the adult mammalian central nervous system was described several years ago [1,2]. Since then, numerous studies have attempted to elucidate the cues, both exogenous and endogenous, that maintain or modify neurogenic activity [3].

The adult rodent hippocampus is capable of maintaining its capacity to generate new neurons. These new cells are generated in the subgranular zone (SGZ) of the dentate gyrus (DG) [4]. Interestingly, proliferative cells have also been described in the hilus [5,6,7,8,9,10,11,12], and these cells have been identified as oligodendrocyte progenitor cells (OPCs) and neural progenitor cells (NPCs) [6]. Furthermore, evidence suggests that these cells can differentiate into microglia and oligodendrocytes, albeit to a limited extent [12]. Their ability to differentiate into neurons is still controversial [5,11], and their rates of cell survival and neuronal differentiation have not been elucidated. Finally, the involvement of the hilar neurogenesis process in hippocampal physiology is unknown, but it may be relevant as hilar proliferation may increase or decrease due to epilepsy or thyroid status, respectively [12,13].

Thyroid hormones (THs) are endogenous signals that play important roles in neurogenesis in the SGZ [14,15]. In this region, THs do not change the proliferative activity of precursor cells [13,16,17,18], but they are necessary for the survival of postmitotic progenitor cells, neuroblasts, and immature granule neurons [13,16,17,18,19,20]. In these latter cells, THs also promote their neuritic growth and stimulate their progression to mature neurons [16,19]. In contrast, evidence concerning the role of THs in the hilar neurogenesis process is limited. THs are known to be required for cell proliferation [13,17], but their effects on the survival and differentiation of hilar postmitotic cells are unknown.

Cell differentiation is a process that involves several stages and culminates in the formation of glial cells or neurons which have the capacity to integrate into circuits that are already present in the nervous system [21,22,23]. There are extrinsic and intrinsic mechanisms that regulate the progression of the adult neural stem lineage, one of which is of particular importance for cellular metabolism, especially energy production, as proliferative cells require more energy [24,25,26]. Because of their well-known effects on cellular metabolism, THs have become important cues. Indeed, several reports have shown the involvement of THs in neuronal or oligodendrocyte differentiation in the subventricular zone, where there is a high rate of oligodendrocyte generation [27,28,29].

The evidence suggests that the hilus harbors progenitor cells and that this region could be considered a potential source of new oligodendrocytes or microglia in the adult brain. However, further research on hilar neurogenesis is needed to characterize other phases of the process and determine the factors that modulate it. The present study aimed to characterize the mitotic activity, cell survival, and neuronal differentiation of hilar neurogenesis under physiological conditions and to determine the effects of hypothyroidism on these cellular events. Finally, comparative evidence is provided with the granular layer (GL) and the SGZ of the DG, a well-established neurogenic zone.

## 2. Materials and Methods

### 2.1. Animals

Male Wistar rats (n = 19) aged 60 days were kept individually in a room with controlled light (12:12) and temperature (21 ± 2 °C). Water and food were always available. Every experimental technique used in this study complies with Mexican legislation (NOM-062-ZOO-1999) and they have all been approved (15 June 2020) by the Animal Care and Use Committee of the Instituto Nacional de Pediatría (Protocol No. 2020/027) and the Ethics Committee of Research on Animal Studies of the Escuela Nacional de Ciencias Biológicas. All possible measures were implemented to reduce the number of animals utilized and to mitigate any discomfort they might have endured.

**Figure 1:**
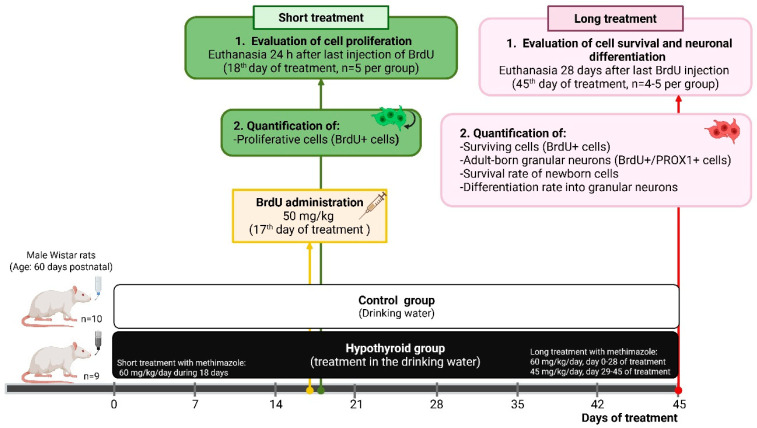
Experimental design. Experimental design showing the experimental groups (control and hypothyroid), as well as the timing of BrdU administration (yellow box) and methodological objectives of the short (green boxes) or long (red boxes) treatment.

The rats were randomly allocated to two experimental groups: the control and hypothyroid groups. The control group received drinking water, whereas the hypothyroid group was treated for 18 days (short treatment) or 45 days (long treatment) with the antithyroid drug methimazole (MMI) dissolved in the drinking water. The short treatment involved the administration of MMI at a dose of 60 mg/kg/day, whereas the long treatment consisted of a 60 mg/kg/day dose from treatment days 0–28, followed by a 45 mg/kg/day reduction from days 29–45, with the objective of preventing the deterioration of the animal’s health (Figure 1). During the exposure period to MMI, the following data were recorded: water intake, rectal temperature, and body weight. Water consumption and body weight data were utilized to calculate the MMI quantity dissolved during the treatment.

The administration of a short MMI treatment was employed to induce hypothyroidism in a more expeditious manner, yet with sufficient severity to allow the cell division of proliferative cells to occur in hypothyroidism conditions. It was necessary that the MMI treatment was administered over a prolonged period of 45 days in order to facilitate the survival of the initial proliferative cells (labeled with BrdU on day 17) and ensure the completion of their neuronal maturation and differentiation. This is due to the fact that it has been documented that these specific cellular processes require a minimum duration of 4 weeks [5,30].

### 2.2. Thyroid Status Assessment

Thyroid status was evaluated indirectly by monitoring rectal temperature and body weight during treatment (thyroid status-dependent variables). Furthermore, in order to corroborate the establishment of hypothyroidism by MMI, triiodothyronine (T_3_) and thyroxine (T_4_) serum concentrations were measured at different times. Blood samples were collected from the tail veins of the rats in both experimental groups on treatment days 0 (basal) and 17 for the short treatment groups and on days 0, 17, and 45 for the long treatment groups. Serum was separated by centrifugation (1095× *g* × 15 min) and stored at −20 °C until analysis. The determination of total T_3_ and T_4_ serum levels was conducted using an immunoassay system employing a commercial kit (DRG Diagnostics, Marburg, Germany). The lowest calibrators were 0.5 ng/mL for the T_3_ assay and 2 μg/dL for the T_4_ assay. The assay sensitivities were 0.2 ng/mL for T_3_ and 0.5 μg/dL for T_4_; such values were assigned to samples whose amounts were below the lowest calibrator.

### 2.3. BrdU Labeling and Tissue Processing

On the 17th day of treatment, the animals in both experimental groups received six injections of BrdU (5-bromo-2′-deoxyuridine, 50 mg/kg), a thymidine analog that is incorporated into DNA during the S phase of the cell cycle [30]. BrdU was dissolved in saline solution (0.9%), and the injections were administered at 2 h intervals (ip). Half of the experimental animals (those in the short treatment group) in the control and hypothyroid groups were euthanized 24 h after the last injection of BrdU (18th day of treatment) with the intention of identifying proliferative cells, whereas the rest of the animals in the long treatment groups continued the study. The animals in the latter group were euthanized 28 days after the last administration of BrdU (45th day of treatment). This was performed to determine cell survival and allow the neuronal differentiation of newborn cells (Figure 1). For euthanasia, the rats were anesthetized with phenobarbital (120 mg/kg) and perfused with 0.9% NaCl followed by 4% paraformaldehyde (PFA) in phosphate-buffered saline (PBS, pH 7.4). The brains were then immersed in a solution of 4% PFA and stored overnight. Following this, they were transferred to sucrose solutions of 10%, 20%, and 30% sucrose, and stored for 24 h in each. Using a cryostat, the dorsal hippocampus (AP) −2.6 to −4.4 mm, according to Paxinos and Watson [31], was serially sectioned to obtain slices that were 20 μm thick. Prior to immunofluorescence processing, slices were placed on gelatinized slides and kept at −60 °C.

### 2.4. Immunofluorescence

For the animals in the short treatment, a single BrdU labeling procedure was conducted. For the animals in the long treatment, BrdU+ and PROX1+ (prospero homeobox protein 1) double labeling was performed (Figure 1). Briefly, the sections were hydrated in 1 × PBS (pH 7.4) and incubated in 1.3 N HCL at 37 °C for 45 min for DNA denaturation. After acid treatment, the slices were immersed in borate buffer (0.1 M, pH 8.5) for 15 min and then permeabilized with Triton X-100 (0.2% in 1 × PBS). The sections were blocked for one hour with a solution containing 0.2% Triton X-100, 2% horse serum, and 1% bovine serum albumin. Then, the samples were subjected to an incubation with an antibody against BrdU (biotinylated mouse IgG, Invitrogen Kit, Waltham, MA, USA) for a duration of 20 h at room temperature (RT). At the end of the incubation period, BrdU staining was performed with FITC–streptavidin (1:50, Invitrogen, Waltham, MA, USA) or DyLight 594–streptavidin (1:100, Vector Laboratories, Newark, CA, USA) for 2 h at RT. Subsequent to the completion of BrdU staining, the tissue underwent processing for double labeling for PROX1. The slices were then subjected to an incubation with an anti-PROX1 antibody (1:1250, rabbit IgG, DakoCytomation, Santa Clra, CA, USA) for 20 h at RT. Finally, secondary anti-rabbit IgG Alexa Fluor 488 (1:100, Life Technologies, Carlsbad, CA, USA) antibody was applied for 2 h at RT.

All slices were mounted using Vectashield supplemented with the nuclear marker DAPI (Vector Laboratories, Newark, CA, USA). The fluorescent signals were detected with a fluorescence microscope (Nikon H6000L, Tokyo, Japan), and the resulting images were processed with Image-Pro Plus version 4.5 software (Media Cybernetics; Rockville, MD, USA). The images were subjected to only general brightness and contrast adjustments, with no other alterations or manipulations being applied to the figures.

### 2.5. Cell Counting

The present investigation was confined to the dorsal area of the DG of the hippocampus; this region was sectioned into slices, and cell counting was carried out in ten slices per rat (200 μm apart). The quantification of positive cells (BrdU+ or BrdU+/PROX1+) was conducted in both brain hemispheres. The total number of cells per rat was determined by calculating the sum of the positive cells identified across ten consecutive slices. The data obtained from various animals within the same experimental group were averaged and presented as a mean value in the graphs. BrdU+ and BrdU+/PROX1+ cells were quantified in three regions of the DG: the GL, SGZ, and hilus. The GL and SGZ are collectively termed the “neurogenic zone” because adult neurogenesis involves dynamic processes that occur in both regions. BrdU+ and BrdU+/PROX1+ cells were also calculated for the neurogenic zone to allow comparisons between the canonic neurogenic region and the hilus. The GL is defined as the region constituted by the cell bodies of granular neurons; the SGZ is characterized as the area occupied by two cell bodies facing up and down from the inferior edge of the GL; and the hilus is identified as the area between both layers of granule cells, excluding the SGZ and the portion of CA3 (refer to the schemes in [6]).

Proliferative cells were identified by the presence of BrdU and subsequently quantified in the experimental groups subjected to short MMI treatment on the 18th day (24 h after BrdU administration). BrdU+ cells found four weeks after BrdU administration were defined as surviving cells, and BrdU+/PROX1+ cells were defined as adult-born granular neurons. These cells were quantified in the long MMI treatment group on the 45th day (Figure 1). The colocalization of BrdU and PROX1 with the nuclear marker DAPI was used to confirm the nuclear location of both proteins.

The rate of cell survival was defined as the ratio of the mean number of surviving cells to the mean number of proliferative cells (data and calculations are available in the Supplementary Material). The percentage of neuronal differentiation was defined as the ratio of adult-born granular neurons among surviving cells for each rat. Cell survival and the neuronal differentiation rate were calculated for the neurogenic zone (GL/SGZ) and each zone of the DG (GL, SGZ, and hilus).

### 2.6. Statistical Analysis

All the data are expressed as the means ± SEMs. The statistical analysis was carried out using SigmaPlot 12.2. Body weight, rectal temperature, serum thyroid hormone levels, and survival data in were analyzed using a repeated measures two-way ANOVA followed by Tukey’s post hoc test. Student’s *t* test or the Mann–Whitney rank sum test was used for cell counting. The Mann–Whitney rank sum test was used for the comparison of percentages.

## 3. Results

### 3.1. Murine Model of Hypothyroidism Induced by Treatment with Methimazole for 18 or 45 Days

**Figure 2:**
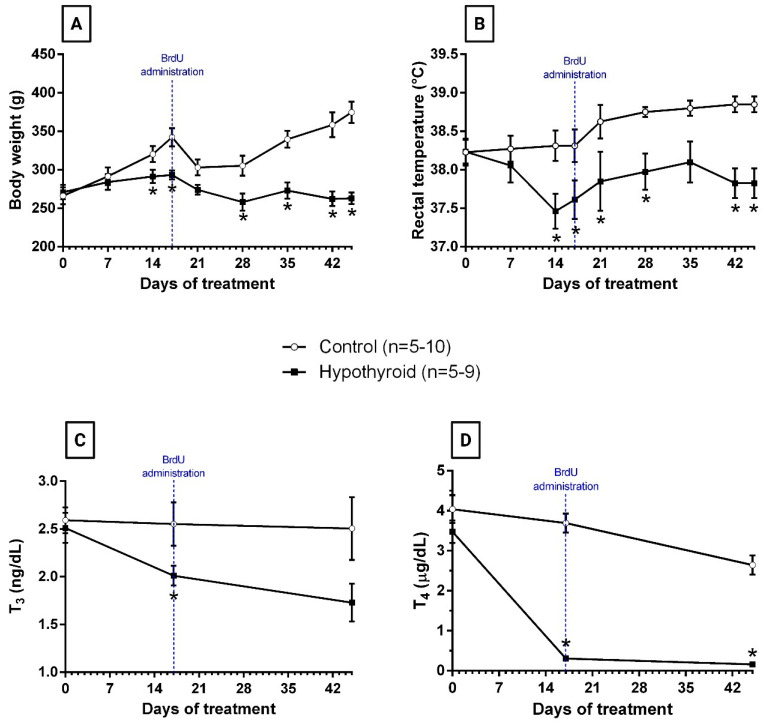
Follow-up of thyroid status. The graphs show the body weight (**A**) and rectal temperature (**B**) over the course of 45 days of treatment in the control group (empty circles) and hypothyroid group (black squares). Serum levels of T_3_ (**C**) and T_4_ (**D**) hormones measured on days 1, 17, and 45 in the control group (empty circles) and hypothyroid group (black squares). The blue line indicates the time of BrdU administration. * *p* < 0.05 versus control at the same time. No significant differences were found in rectal temperature and serum levels of T_3_ and T_4_ among the times before and after BrdU administration in the control oy hypothyroid groups.

Hypothyroidism was induced by the administration of the antithyroid drug MMI in the drinking water. Compared with the control treatment, the administration of this drug resulted in lower body weights and rectal temperatures from the 14th day of treatment until the end of the experiment (Figure 2A,B). The hypothyroid state was confirmed by measuring the serum levels of thyroid hormones. A significant reduction in serum T_3_ levels was observed in MMI-exposed rats compared with control rats on day 17 of treatment. Although this reduction was not significant on day 45 of treatment, lower serum levels were observed in the hypothyroid group on this day (Figure 2C). Finally, significant reductions in serum T_4_ levels were detected in MMI-exposed rats on days 17 and 45 of treatment compared with those in the control group (Figure 2D).

In this study, rats were exposed to MMI for different periods of time: a short period of 18 days (MMI dose: 60 mg/kg/day) and a longer period of 45 days (MMI doses: 60 mg/kg/day and then 45 mg/kg/day). The results demonstrated that administration of MMI for 18 days was sufficient to induce hypothyroidism, as evidenced by lower body weight, reduced rectal temperature, and decreased serum T_3_ and T_4_ levels on day 17 of antithyroid treatment compared with those of the controls (Figure 2A–D). This hypothyroid state was sustained despite the reduction in the MMI dose, as evidenced by the lack of significant differences in the thyroid status variables before (14th, 17th, 21st, and 28th days of treatment) and after (35th, 42nd, and 45th days of treatment) the dose change in the hypothyroid group (Figure 2A–D). These results support that prolonged treatment with two doses of MMI was effective in inducing hypothyroidism and maintaining it until the end of the experiment.

### 3.2. Cell Proliferation, Cell Survival, and Neuronal Differentiation in the Hilus Under Physiological Conditions

**Figure 3:**
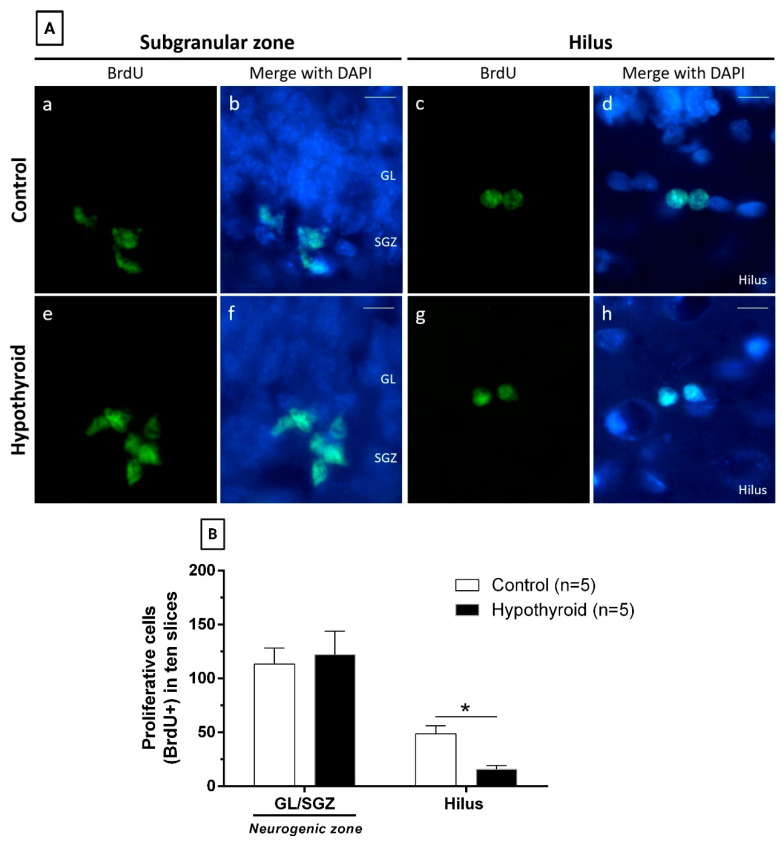
Hypothyroidism decreases the number of proliferative cells in the hilus of the dentate gyrus. (**A**) Representative photomicrographs displaying proliferative cells (BrdU positive cells) located in the subgranular zone (SGZ) or the hilus from two distinct experimental groups: control (**a**–**d**) and hypothyroid (**e**–**h**). This study was conducted on day 18 of treatment. Green: BrdU and blue: nuclear marker DAPI. Scale bar, 20 μm. (**B**) Quantification of proliferative cells from control and hypothyroid rats in the neurogenic zone (granular layer (GL) and subgranular zone (SGZ)) and hilus of the dentate gyrus on day 18 of treatment. BrdU: 5-bromo-2′-deoxyuridine. * *p* < 0.05 versus control in the hilus.

Proliferative cells (BrdU-positive cells) were localized in the neurogenic zone (GL/SGZ) and hilus of adult control rats on day 18 of treatment (Figure 3A(a–d)). We found that the hilus contained an average of 48.6 proliferative cells in ten hippocampal slices (width: 20 µm), and this number was significantly lower than that in the neurogenic zone (*p* = 0.005) (Table 1). After 28 days of BrdU labeling, the numbers of surviving cells were similar in both regions (Table 1). However, the survival rate of newborn hilus cells was 18.9%, which was higher than that in the neurogenic zone (10.3%) (Table 1). In terms of neuronal differentiation, the number of adult-born granular neurons was significantly lower in the hilus than in the neurogenic zone (1.6 vs. 7.2; *p* = 0.003), which was consistent with a lower neuronal differentiation rate in the hilus (19%) than in the neurogenic zone (65.7%) (*p* = 0.03) (Table 1).

### 3.3. Hypothyroidism-Related Changes Depend on the Region of the DG

Proliferative cells were also found in the neurogenic zone and hilus of adult hypothyroid rats (Figure 3A(e–h)). The analysis of cell counting indicated that hypothyroidism led to a decrease in the number of proliferative cells exclusively within the hilus region (control, 48.6 ± 7.5; hypothyroid, 15.8 ± 3.4; *p* = 0.004), whereas the number of these cells was not affected in the neurogenic zone (control, 113.2 ± 15.0; hypothyroid, 122 ± 22.0; Figure 3B). Similar results were previously reported with longer-term hypothyroidism treatment [13].

**Figure 4:**
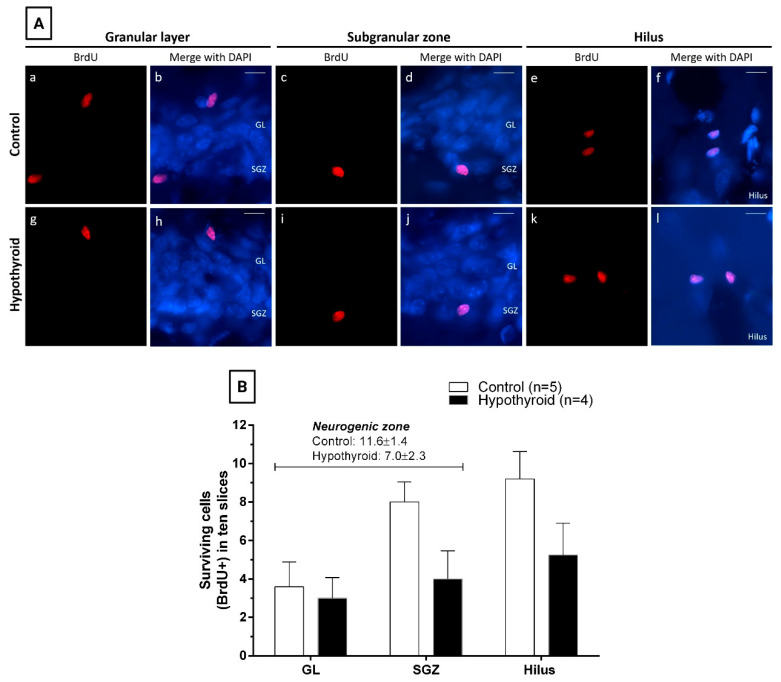
Adult-onset hypothyroidism does not affect the number of surviving cells in the granular layer, subgranular zone, or hilus of the dentate gyrus. (**A**) Representative photomicrographs displaying surviving cells (BrdU positive cells) located in the granular layer (GL), subgranular zone (SGZ), or the hilus from two distinct experimental groups: control (**a**–**f**) and hypothyroid (**g**–**l**). This study was conducted on day 45 of treatment (28 days after the last BrdU injection). Red: BrdU and blue: nuclear marker DAPI. Scale bar, 20 μm. (**B**) Quantification of surviving cells from control and hypothyroid rats in the neurogenic zone (GL and SGZ, cursive), as well as in the GL, SGZ, and hilus of the dentate gyrus (bars) at the 45th treatment day. BrdU: 5-bromo-2′-deoxyuridine.

To determine the effect of hypothyroidism on cell survival, BrdU-positive cells were quantified 28 days after BrdU administration in the GL, SGZ, and hilus of the DG (Figure 4A). No difference was found in the quantification of surviving cells between control and hypothyroid rats in the neurogenic zone (Figure 4B). Similarly, the data revealed no differences in the number of surviving cells between control and hypothyroid animals in the three zones examined, although the numbers were always lower in the hypothyroid group (GL: control 3.6 ± 1.3 and hypothyroid 3.0 ± 1.1; SGZ: control 8.0 ± 1.0 and hypothyroid 4.0 ± 1.5; hilus: control 9.2 ± 1.4 and hypothyroid 5.3 ± 1.7) (Figure 4B).

**Figure 5:**
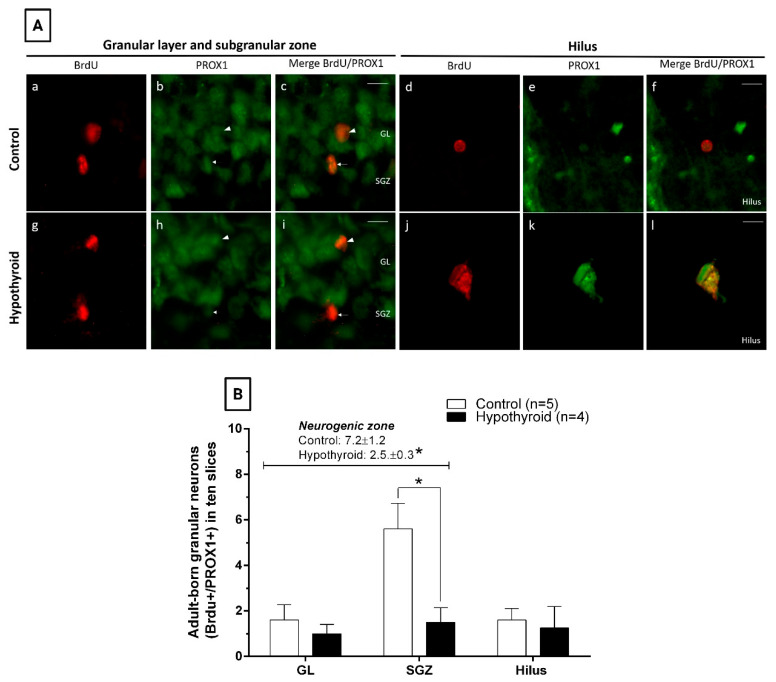
Adult-onset hypothyroidism selectively reduces adult-born granular neurons in the neurogenic zone, exerting a specific effect on the ZSG. (**A**) Representative photomicrographs showing adult-born granular neurons (BrdU+/PROX1+ cells) located in the granular layer (GL, arrowheads), subgranular zone (SGZ, arrows), or hilus from control (**a**–**f**) and hypothyroid (**g**–**l**) adult rats on the 45th treatment day (28 days after the last BrdU injection). Red: BrdU and green: PROX1. Scale bar, 20 μm. (**B**) Quantification of adult-born granular neurons from control and hypothyroid rats in the neurogenic zone (GL and SGZ, cursive), as well as in the GL, SGZ, and hilus of the dentate gyrus (bars) at the 45th treatment day. BrdU: 5-bromo-2′-deoxyuridine. PROX1: Prospero-related homeobox 1. * *p* < 0.05 versus control in the SGZ.

Furthermore, the impact of hypothyroidism on neuronal differentiation was analyzed through the quantification of adult-born granular neurons positive for BrdU and PROX1 markers located in the GL, SGZ, and hilus of the DG (Figure 5A). The results revealed that hypothyroidism selectively reduced the number of adult-born granular neurons in the neurogenic zone (*p* = 0.016), with a specific effect on the SGZ (SGZ: control 5.6 ± 1.1 and hypothyroid 1.5 ± 0.6, *p* = 0.016) (Figure 5B). Conversely, no alterations were observed in either the GL or the hilus of hypothyroid rats in comparison with those of the control group (GL: control, 1.6 ± 0.7; hypothyroid, 1.0 ± 0.4; hilus: control, 1.6 ± 0.5; and hypothyroid, 1.3 ± 0.9) (Figure 5B).

**Figure 6:**
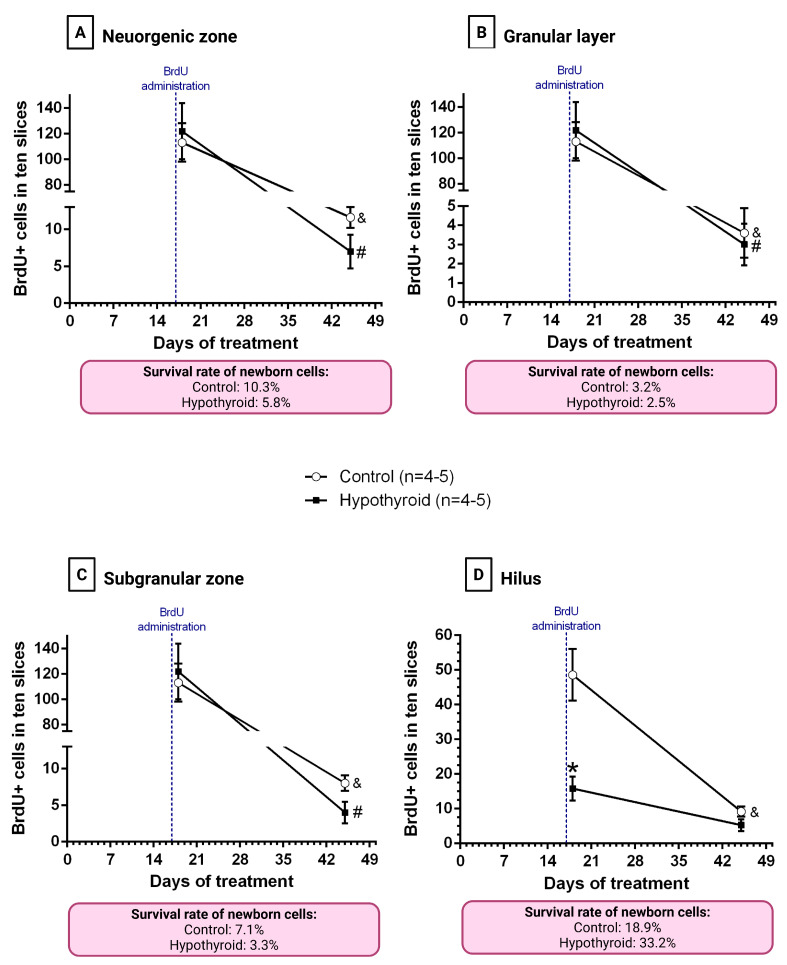
Adult-onset hypothyroidism affects the cell survival rate depending on the region of the dentate gyrus. The plots show the number of BrdU+ cells quantified 24 h (proliferative cells, day 18 of treatment) or 28 days (surviving cells, day 45 of treatment) after BrdU administration in the neurogenic zone (**A**), the granular layer (**B**), the subgranular zone (**C**), and the hilus (**D**), from the control group (empty circles) and hypothyroid group (black squares). Red boxes: percentages of newborn cells that survive from control and hypothyroid adult rats in the neurogenic zone (**A**), granular layer (**B**), subgranular zone (**C**), and hilus (**D**). The blue line indicates the time of BrdU administration. &: *p* < 0.05 vs. control group on day 18 of treatment, #: *p* < 0.05 vs. hypothyroid group on day 18 of treatment, *: *p* < 0.05 vs. control group on day 18 of treatment. The data and the procedure for calculating the percentages are described in the Section 2 and in the “Supplementary Material”.

The cell survival rate of newborn cells was calculated as the ratio of the mean number of surviving cells to the mean number of proliferative cells. Firstly, we analyzed the number of BrdU+ cells at each time point, and then we calculate the cell survival rate (Figure 6A–D). With the exception of the hilus in the hypothyroid group, all studied regions showed a significant reduction in the number of surviving cells (BrdU+ quantified 28 days after BrdU treatment) compared to the initial number (BrdU+ cells quantified 24 h after BrdU treatment) in the control and hypothyroid groups (*p* < 0.05) (Figure 6A–D). In the neurogenic zone, the cell survival rate was 10.3%, with 3.2% in the GL group and 7.1% in the SGZ in the control group. Hypothyroidism led to a decrease in the cell survival rate to 5.8% in the neurogenic zone, with 2.5% and 3.3% recorded in the GL and SGZ, respectively (Figure 6A–C). Interestingly, the effect of hypothyroidism was the opposite in the hilus as MMI treatment increased the percentage of surviving hilus cells to 33.2% in the hypothyroid rats (control 18.9%, hypothyroid 33.2%, Figure 6D).

**Figure 7:**
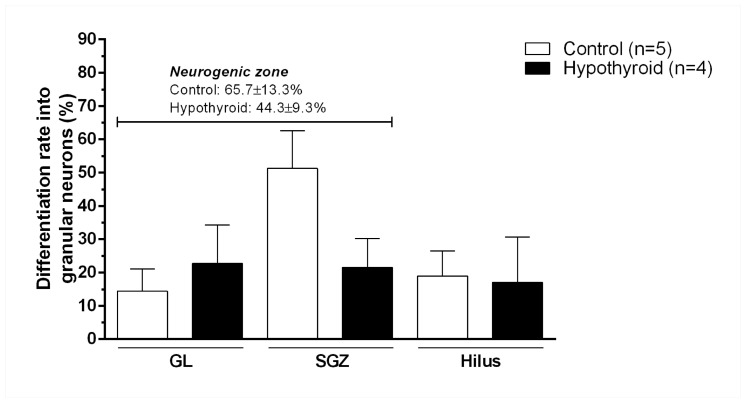
Adult-onset hypothyroidism reduced the neuronal differentiation rate in the neurogenic zone, but it was not statistically significant. Percentage of cells that became granular neurons from control and hypothyroid adult rats in the granular layer (GL), subgranular zone (SGZ), and hilus. Additionally, the total rate of neuronal differentiation in the neurogenic zone is shown in cursive. The procedure for calculating the percentages is described in Section 2.

With respect to the differentiation rate into granular neurons, the neuronal differentiation rate was 65.7% in the neurogenic zone of the control group, with 14.4 ± 6.7% and 51.3 ± 11.4% in the GL and SGZ, respectively. Hypothyroidism reduced the neuronal differentiation rate to 44.3% in the neurogenic zone, with a specific effect on the SGZ (GL: 22.8 ± 11.5, SGZ: 21.6 ± 8.6). However, these alterations were not statistically significant. Finally, the neuronal differentiation rates in the hilus were similar between the control and hypothyroid groups (control, 19 ± 7.6; hypothyroid, 17.1 ± 13.6; Figure 7).

## 4. Discussion

The hilus contains a population of proliferative cells [5,6,7,8,9,10,11,12]. These cells can modify their mitotic activity under the influence of various factors [8,9,11,32]. However, other cellular events, such as cell survival and neuronal differentiation under physiological conditions, remain to be explored more extensively. In addition, the involvement of THs in adult neurogenesis in the SGZ of the DG has been widely studied [33,34,35], but their role in neurogenesis in the hilus is still poorly explored.

In this work, we characterize the mitotic activity, cell survival, and neuronal differentiation in the hilus compared with the neurogenic zone under physiological conditions in adult Wistar rats. Furthermore, the results show that the involvement of THs in cells born in the adult brain differs between the hilus and the neurogenic zone of the DG of the hippocampus.

### 4.1. Adult-Onset Hypothyroidism by Methimazole Administration for 18 or 45 Days in Wistar Rats

In this study, hypothyroidism was provoked by the oral treatment of MMI. MMI reduces thyroid hormone synthesis in the thyroid gland by inhibiting the activity of the enzyme thyroid peroxidase (TPO); this antithyroid drug competes with thyroglobulin-bound tyrosine residues and diverts oxidized iodide away from hormone synthesis [36,37,38]. In addition, in vitro studies have shown that methimazole reacts with molecular iodine to form 1-methylimdazole, suggesting that this drug may reduce the iodine availability for the iodination of tyrosine residues [39]. As MMI accumulates specifically in the thyroid gland [40], the observed neurogenic effects must be a consequence of reduced TH levels and not a direct effect of MMI on the brain.

Unlike previous studies (e.g., [41]), MMI treatment was performed for 18 or 45 days in adult Wistar rats. MMI was administered for 18 days (dose: 60 mg/kg/day) to induce hypothyroidism in a shorter time but with sufficient severity to ensure that central TH levels had decreased (as reported by [42]) when BrdU was administered on day 17. Our results confirmed that this short MMI treatment led to the onset of hypothyroidism, characterized by reduced body weight and rectal temperature in line with the function of THs in thermoregulation and appetite control, as previously reported [43,44,45,46]. In addition, the short MMI treatment induced low serum levels of T_3_ and T_4_, with T_4_ levels decreasing more than T_3_ levels, possibly because T_3_ is synthesized from T_4_ in peripheral tissues. However, because T_4_ is the main source for T_3_ synthesis in the brain [47], we argue that the availability of THs is reduced in the hippocampus; consequently, the proliferative cells underwent cell division and started their neuronal differentiation in a hypothyroid state.

MMI treatment for 45 days effectively induced overt hypothyroidism in the rats, with changes in thyroid status-dependent variables similar to those observed in the short-term treatment group. In this case, the MMI treatment was extended for 28 additional days, meaning that the surviving cells and the adult-born granular neurons started and finished their cell differentiation in a hypothyroid state. To avoid excessive adverse peripheral effects due to hypothyroidism, and in accordance with the 3Rs principle [48], long-term treatment with MMI was refined by reducing the dose of MMI from 60 mg/kg/day to 45 mg/kg/day from the 28th to 45th day of treatment. This refinement had no significant effect on the existing hypothyroidism, as there were no significant differences in thyroid status-dependent variables between the days before and after the dose change. However, it was beneficial for the experimental animals as they maintained the same body weight and did not show an excessive reduction in rectal temperature during the MMI treatment.

### 4.2. Hilus of the DG: Proliferation, Survival, and Neuronal Differentiation Under Physiological Conditions

Consistent with the findings of other authors [5,10,11] and with previous reports from our laboratory [6,13], we found that the number of proliferative cells in the hilus was lower than that observed in the neurogenic zone. The presence of hilar proliferative cells has long been described [5,7,8,9,10,11,12,13], and they are mainly OPCs and NPCs [6]. These cells are responsive to pathological events as their proliferation rate is affected by hypothyroidism or seizures [12,13]. Despite the lack of a neurogenic niche, the astrocytes [49] and microvessels [50] located in the hilus may be responsible for controlling hilar proliferation and the differentiation of progenitor cells. Although it has been proposed that neurogenesis does not generate oligodendrocytes in vivo [51,52], OPCs are widely distributed in the central nervous system, including the hippocampus [6,53,54], where they are able to differentiate into oligodendrocytes in adult mice [55].

Similarly to that in the neurogenic zone, the number of BrdU+ cells observed in the hilus 28 days after the last BrdU injection decreased compared with that observed 24 h after BrdU treatment. This pattern has been previously reported by Wennström et al. [11] and could be attributed to the loss of BrdU labeling due to the mitotic activity of proliferative cells. This is related mainly to the number of dead cells undergoing apoptosis, which limits the survival of newborn cells [56].

To the best of our knowledge, this is the first report of the rates of cell survival and neuronal differentiation in the hilus. The cell survival rate in the hilus was found to be almost twice as high (18.9%) as that in the neurogenic zone (10.3%). Conversely, the rate of differentiation into granular neurons was significantly lower in the hilus (19%) than in the neurogenic zone (65.7%). The presence of newborn neurons in the hilus has also been demonstrated by double labeling with [^3^H]thymidine and the neuron-specific enolase (NSE) marker [5,32]. Given the evidence that the hilus contains NPCs [6], it can be hypothesized that newborn granular neurons in the hilus may be derived from these hilar NPCs, suggesting the potential for the generation of other types of neurons in this region. We cannot determine whether newborn neurons from the hilus are ultimately integrated into the GL of the DG, but they could be important reservoirs that provide mature neurons to maintain hippocampal functionality. However, these newborn neurons may be counterproductive, as those ectopic hilar newborn neurons may be associated with the formation of aberrant circuitry leading to seizures [57].

### 4.3. Differential Effects of Hypothyroidism in the Neurogenic Zone and Hilus

Hypothyroidism provoked differential effects that were dependent on the hippocampal region. Hypothyroidism causes a significant decrease in the number of proliferative cells in the hilus without affecting the neurogenic zone. Our results do not provide evidence for the possible mechanisms by which THs modify proliferation. However, there is evidence that THs regulate the expression of the sonic hedgehog (Shh) signaling pathway in the adult DG [58]. Shh has been shown to be involved in the proliferation, maturation, and differentiation of neuronal and oligodendrocyte precursors during neurodevelopment and adult neurogenesis [59,60,61,62].

Interestingly, we also observed a differential effect between the hilus and DG in the survival rate of newborn neurons and in the rate of their differentiation into granular neurons. Hypothyroidism provoked a greater percentage of cell survival in the hilus and the opposite effect in the neurogenic zone. Furthermore, cell fate also exhibited marked differences as the number of adult-born granular neurons was significantly reduced in the neurogenic zone by MMI treatment, without alterations in the hilus. These results show that the progenitor cell population behaves differently in the two regions, in addition to the fact that THs have different effects in each region. In fact, the proliferative population is different between the two regions. Although the hilus has a lower number of proliferative cells, it harbors OPCs and NPCs in equal proportions, whereas the neurogenic zone has five times more NPCs than OPCs [6]. The idea that THs have differential effects depending on the region has been reported previously; several reports have associated hypothyroidism with decreased progenitor cell death in the subventricular zone and increased progenitor cell death in the SGZ, suggesting that thyroid hormone effects on adult progenitors may have niche-specific effects (for review, see [34]).

The cellular mechanisms by which THs regulate stem cell fate are still poorly understood. Recently, the importance of monocarboxylate transporter 8 (MCT8) [63] and the nuclear receptor TRα1 [18] for cell cycle exit and neuronal differentiation has been described. Another possibility for stem cell fate may be related to metabolic energy availability. The importance of mitochondria for neuronal differentiation has already been described [64]. It is well known that THs are important for mitochondrial activity [65,66]; thus, altering TH levels could alter stem cell fate choices. Recently, mitochondrial metabolism has been shown to play an important role in promoting the acquisition of neuronal fate choices [67]. The change from proliferative activity to neuronal differentiation is associated with a switch from glycolytic to oxidative phosphorylation for energy needs [25,68,69]. The effects of THs at the cellular level may involve multiple modes of action as THs alter gene expression, which in turn alters mitochondrial metabolism [70].

### 4.4. Two Populations of Proliferative Cells in the DG of the Adult Brain

The results of the present work describe the effects of hypothyroidism on cell proliferation, cell survival, and neuronal differentiation in the hilus and neurogenic zone of the adult rat brain. The results reveal that hypothyroidism had a differential effect on the regions studied. In the neurogenic zone, thyroid deficiency does not affect cell proliferation; however, it significantly decreases the rate of cell survival and the number of adult-born granular neurons. In the hilus, hypothyroidism decreases cell proliferation and increases the rate of cell survival without affecting neuronal differentiation. The findings of this study are consistent with those of other investigations reporting a differential response between the neurogenic zone and hilus induced by hormonal factors (corticosterone levels) [32], pharmacological factors (stimulant drugs) [8], biological factors (animal age) [9], and pathological factors (seizures) [11]. These findings, together with the characterization of the pool of proliferative cells in the neurogenic zone and hilus [6], support that the DG possesses two populations of proliferative cells whose cell cycle, survival, and neuronal differentiation are independently modified by several factors. Additionally, the proliferative cells in the hilus may represent a source of new cells with possibly different physiological functions.

## 5. Conclusions

Our data confirm that the hilus of the DG harbors proliferative cells. Compared with the neurogenic zone, the hilus exhibited a reduced number of proliferative cells and a lower neuronal differentiation rate, with an increased cell survival rate.

THs play a pivotal role in neurogenesis within the hilus, given that hypothyroidism provoked decreased cell proliferation and an increased rate of cell survival. Notably, hypothyroidism exerts differential effects on the hilus and neurogenic zones. This finding suggests that the DG comprises two distinct populations of proliferative cells, each with a cellular physiology that is independently modified by the THs.

## Figures and Tables

**Table 1:** Mitotic activity, cell survival, and neuronal differentiation in the neurogenic zone and hilus of the DG under physiological conditions.

	Region of the Dentate Gyrus		*p*
	Neurogenic Zone (GL and SGZ)	Hilus	
Number of proliferative cells (BrdU+ cells, 24 h after last BrdU injection)	113.2 ± 15.0	48.6 ± 7.5	0.005
Number of surviving cells (BrdU+ cells, 28 days after last BrdU injection)	11.6 ± 1.4	9.2 ± 1.4	ns
Survival rate of newborn cells	10.3%	18.9%	
Number of adult-born granular neurons) (BrdU+/PROX1+ cells, 28 days after last BrdU injection	7.2 ± 1.2	1.6 ± 0.5	0.003
Differentiation rate into granular neurons	65.7 ± 13.3%	19 ± 7.6%	0.03

Abbreviations: GL: granular layer, SGZ: subgranular zone, BrdU: 5-bromo-2′-deoxyuridine, PROX1: Prospero-related homeobox 1, ns: not significant.

## Data Availability

The original contributions presented in this study are included in the article or Supplementary Material. Further inquiries can be directed to the corresponding author.

## Supplementary Materials

The following supporting information can be downloaded at: https://www.mdpi.com/article/10.3390/cells14141112/s1, Table S1: Percentage of cell survival.
