# Supplementary material for: Mitotic Activity, Cell Survival, and Neuronal Differentiation in the Hilus of the Dentate Gyrus Under Physiological and Hypothyroid Conditions in Adult Wistar Rats
Source: Cells. 2025 Jul 19;14(14):1112. doi: 10.3390/cells14141112 (PMC12293619; doi:10.3390/cells14141112)
Supplement: Supplementary file 1 [file cells-14-01112-s001.zip › cells-3725106-supplementary.pdf]

Supplementary material

Table S1. Percentage of cell survival.

	Neurogenic zone (GL and SGZ)				Hilus	
	Control		Hypothyroid (Hyp)		Control	Hypothyroid (Hyp)
Proliferative cells (mean)[a]	113.2		122.0		48.6	15.8*
	GL		SGZ		Hilus	
	Control	Hyp	Control	Hyp	Control	Hyp
Surviving Cells (mean)[b]	3.6	3.0	8.0	4.0	9.2	5.3
Survival rate of newborn cells (%)	3.2	2.5	7.1	3.3	18.9	33.2

[a]BrdU+ cells at 17th day (Figure 2B). [b]BrdU+ cells at 45th day (Figure 3B). GL: Granular layer, SGZ: Subgranular zone. *:p<0.05 respect his own control.
